# Selective Detection of Target Volatile Organic Compounds in Contaminated Humid Air Using a Sensor Array with Principal Component Analysis

**DOI:** 10.3390/s17071662

**Published:** 2017-07-19

**Authors:** Toshio Itoh, Takafumi Akamatsu, Akihiro Tsuruta, Woosuck Shin

**Affiliations:** National Institute of Advanced Industrial Science and Technology (AIST), Shimo-shidami, Moriyama-ku, Nagoya 463-8560, Japan; t-akamatsu@aist.go.jp (T.A.); a.tsuruta@aist.go.jp (A.T.); w.shin@aist.go.jp (W.S.)

**Keywords:** metal oxide, tin oxide, cerium oxide, principal component analysis, indoor air contamination

## Abstract

We investigated selective detection of the target volatile organic compounds (VOCs) nonanal, *n*-decane, and acetoin for lung cancer-related VOCs, and acetone and methyl *i*-butyl ketone for diabetes-related VOCs, in humid air with simulated VOC contamination (total concentration: 300 μg/m^3^). We used six “grain boundary-response type” sensors, including four commercially available sensors (TGS 2600, 2610, 2610, and 2620) and two Pt, Pd, and Au-loaded SnO_2_ sensors (Pt, Pd, Au/SnO_2_), and two “bulk-response type” sensors, including Zr-doped CeO_2_ (CeZr10), i.e., eight sensors in total. We then analyzed their sensor signals using principal component analysis (PCA). Although the six “grain boundary-response type” sensors were found to be insufficient for selective detection of the target gases in humid air, the addition of two “bulk-response type” sensors improved the selectivity, even with simulated VOC contamination. To further improve the discrimination, we selected appropriate sensors from the eight sensors based on the PCA results. The selectivity to each target gas was maintained and was not affected by contamination.

## 1. Introduction

Human breath includes many volatile organic compounds (VOCs) that can be used as biomarkers for diseases. For example, breath exhaled from diabetes patients includes high concentrations of acetone and methyl *i*-butyl ketone (MiBK) [1], and breath exhaled from lung cancer patients includes higher concentrations of *n*-decane, nonanal, and acetoin than controls [2,3,4]. Therefore, breath-monitoring methods are desirable as diagnostic tools because they are fast and non-invasive diagnostic methods [5]. Consequently, many researchers have attempted to develop breath-monitoring systems [6,7,8].

One possible breath-monitoring system uses semiconductor metal oxide (MO*_x_*) VOC sensors that were developed for a wide range of applications, including indoor air quality monitoring [9,10,11,12,13,14,15], mouth odor monitoring [16,17,18,19], and human health diagnosis [20,21,22,23,24]. In all cases, the selectivity of the gas sensor should be analyzed precisely due to the presence of potentially interfering gases. The selectivity of MO*_x_* sensors can be roughly controlled by adding noble metal catalysts [25]. Alternatively, a sensor array can be analyzed with statistical methods, such as principal component analysis (PCA) [20,21,26,27]. In one study, a screening apparatus was developed that included a sensor array with a solid phase microextraction (SPME) fiber as a VOC-condensing unit [20,21,28,29]. Some VOCs related to several diseases have been detected in exhaled breath at the ppb level [3], which is typically too low for detection by MO*_x_* sensors. The SPME fiber has the advantage of condensing VOCs from breath samples into gas bags to directly expose them to the sensor array. Byun et al. have reported a screening apparatus using a sensor array of four TGS series sensors (TGS 2600, 2610, 2610, and 2620; Figaro Engineering Inc., Minoh, Japan) and a custom-built MO_x_ sensor with an automated SPME desorption system for selectivity to lung cancer-related VOCs [20,21]. However, the sensor signals for target VOCs are also affected by the contamination of VOCs from ambient indoor air. To overcome this problem, the indoor air could be purified and maintained in the sensor chamber during the desorption of VOCs from the SPME fiber. In this case, however, the screening apparatus needs to be equipped with a complicated system, such as a delicate purification system for indoor air, a flow system for purified air into the sensor chamber, and an airtight injection joint for the SPME fiber. Therefore, we have investigated reducing the influence of indoor air contamination by selecting appropriate sensors from the sensor array based on statistical analysis.

In this study, we investigated the effects of contamination on the monitoring of lung cancer- and diabetes-related VOCs under standard humid conditions (60% relative humidity (RH)). The European Commission Joint Research Centre Environmental Institute and Ministry of Health, Labour and Welfare, Japan, have published lists of VOCs that should be monitored for protecting human health [30,31]. We prepared mixtures of 31 VOCs to simulate possible indoor air contamination [15] according to the lists and definitions of VOCs from an international standard organization [32]. In our previous study, to simulate the diagnostic process, we used four TGS sensors defined by Byun et al. [20,21], and two Pt, Pd, and Au-loaded SnO_2_ (Pt, Pd, Au/SnO_2_) sensors in dry air [27]. The two Pt, Pd, Au/SnO_2_ sensors were developed for the detection of low concentrations of VOCs [25,33], including nonanal [24]. These six sensors are classified as “grain boundary-response type” MO_x_ sensors, e.g., SnO_2_. At low VOC concentrations, oxygen adsorbed from air on the surface removes electrons from the MO_x_ conduction band and results in an electron-depleted layer, which acts as a potential barrier between neighboring grains [34,35]. At high VOC concentrations, the VOCs are oxidized by adsorbed oxygen, reducing the potential barrier, so that the concentration of VOCs can be analyzed by monitoring the electrical resistance. However, the described sensor array including the six sensors cannot discriminate lung cancer- and diabetes-related VOCs under humid conditions, even using PCA. Adsorbed water molecules block oxygen from adsorbing onto surface sites and decrease the intensity of the resistance change induced by oxidized VOCs. In this study, we used both “bulk-response type” sensors as well as “grain boundary-response type” sensors. The “bulk-response type” sensors, such as CeO_2_ and Ce_1−*x*_Zr*_x_*O_2_ [36,37], possess high oxygen diffusion coefficients to provide lattice oxygen for oxidizing VOCs. Therefore, because the “bulk-response type” sensors would likely be less affected by humidity, we added them into the sensor array to improve the performance. In addition, we investigated the use of eight sensors for monitoring lung cancer-related VOCs (*n*-decane, nonanal, and acetoin) and diabetes-related VOCs (acetone and MiBK) with simulated indoor air at a standard relative humidity. 

## 2. Materials and Methods 

### 2.1. Gas Sensors

**Figure 1:**
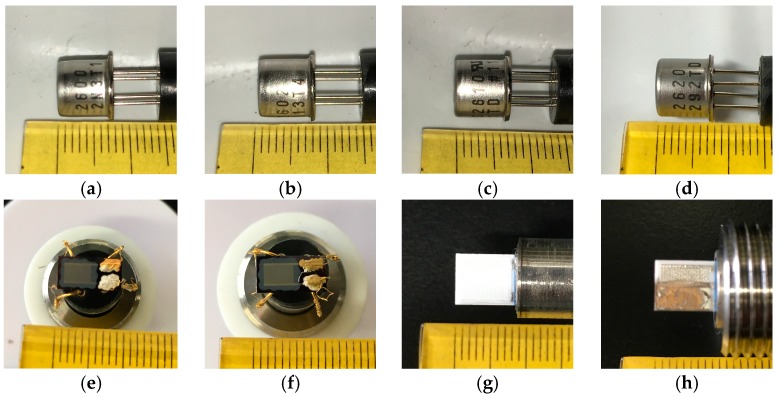
Photos of all sensors: (**a**) TGS 2600; (**b**) TGS 2602; (**c**) TGS 2610; (**d**) TGS 2620; (**e**) #31b; (**f**) #33b; (**g**) No. 9; and (**h**) No. 71.

We selected four commercially available semiconductor metal oxide sensors (TGS 2600, 2602, 2610, and 2620; Figaro Engineering Inc., Minoh, Japan), two semiconductor Pt, Pd, Au/SnO_2_ sensors, and two semiconductor Zr-doped CeO_2_ sensors. The Pt, Pd, Au/SnO_2_ sensors and the Zr-doped CeO_2_ sensors were prepared according to previous reports [24,38]; photos of all sensors are shown in Figure 1, and the details of the sensors are summarized in Table 1.

Pt, Pd, and Au aqueous colloid suspensions (particle size: 2–4 nm; Tanaka Kikinzoku Kogyo, Tokyo, Japan) were added to SnO_2_ powder (particle size: around 100 nm; Aldrich, St. Louis, MO, USA). The Pt, Pd, and Au content was 1 wt % of SnO_2_. The mixture was stirred, dried, and subsequently heated at 400 °C for 2 h in air. The resulting powder was added to an ethylcellulose-type organic dispersant to obtain a paste with powder/vehicle ratios of 1/16 and 1/8. The pastes were subsequently applied to surface-oxidized silicon substrates with platinum comb-type electrodes using a Musashi Engineering FAD-320s dispenser. The substrates were dried at 80 °C for 2 h and subsequently annealed at 500 °C for 2 h in ambient air. The two Pt, Pd, Au/SnO_2_ sensors had a thickness of 2.8 and 4.6 μm for the 1/16 and 1/8 powder/vehicle ratio pastes, and were referred to as “#33b” and “#31b,” respectively.

Cerium nitrate pentahydrate (Kojundo Chemical Laboratory, Sakado, Japan) and zirconyl nitrate dihydrate (Wako Pure Chemical Industries, Osaka, Japan) were dissolved in distilled water to obtain solutions containing 90 mmol/L Ce^3+^ and 10 mmol/L Zr^4+^, respectively. Aqueous ammonia (25%) was then added dropwise to the aqueous solution to form a white precipitate. The precipitate was filtered, and mixed with commercialized carbon powder (Mitsubishi Chemicals, Tokyo, Japan) using a Keyence HM-500 hybrid mixer to give a precipitate/carbon powder weight ratio of 75:11. The mixture was then dried at 70 °C overnight and annealed at 900 °C for 2 h to give the final product Ce_0.9_Zr_0.1_O_2_ (CeZr10) as a fine powder. The powder was then added to an ethylcellulose-type organic dispersant to obtain a paste with a powder/vehicle ratio of 1/4.

The CeZr10 paste was screen-printed onto the electrode of alumina substrates with a platinum comb-type electrode using a New Long Seimitsu Kogyo LS-150 screen printer, dried at 150 °C for 15 min, and then subjected to four print/dry cycles. The resulting substrate was calcined at 500 °C for 5 h and fired at 1100 °C for 2 h to obtain the CeZr10 thick film sensor, referred to as “No. 9”. An additional Zr-doped CeO_2_ sensor was prepared in a similar manner. Powder of γ-alumina prepared as described in our previous report [39] was also added to an ethylcellulose-type organic dispersant to obtain a paste with a powder/vehicle ratio of 1/2. The γ-alumina paste was screen-printed onto the CeZr10 thick film, and the print/dry process was repeated three times in a similar manner to the CeZr10 thick film. Subsequently, the CeZr10 paste was screen-printed onto the γ-alumina thick film, underwent four print/dry cycles, and was calcined at 800 °C for 2 h. A Pt colloid suspension (particle size: 2 nm, Tanaka Kikinzoku Kogyo, Tokyo, Japan) was added onto the surface of the resulting CeZr10/Al_2_O_3_/CeZr10 layered thick film and dried at 70 °C to obtain a Pt content of 3 wt % on the upper CeZr10 thick film. This Zr-doped CeO_2_ sensor (CeZr10/Al_2_O_3_/Pt-CeZr10) was referred to as “No. 71”. 

### 2.2. Preparation of Target VOCs

**Figure 2:**
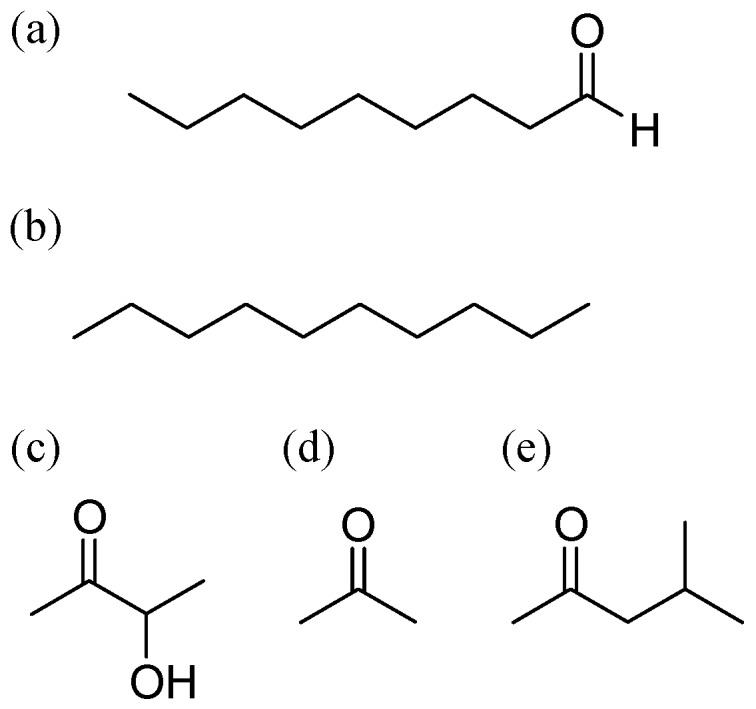
Structural formulas of the target gases: (**a**) nonanal; (**b**) *n*-decane; (**c**) acetoin (3-hydroxy-2-butanone); (**d**) acetone; (**e**) methyl *i*-butyl ketone (MiBK; 2-butanone).

The molecular structures of the target VOCs are shown in Figure 2. The target gases of nonanal (Tokyo chemical industry, Tokyo, Japan), *n*-decane (Tokyo chemical industry, Tokyo, Japan), and acetoin (Tokyo chemical industry, Tokyo, Japan) were generated from their solvents by a Gastec Permeator PD-1B gas generator. Target gases of acetone and MiBK were prepared using cylinders of standard gases (50 ppm in nitrogen; Sumitomo Seika Chemicals, Osaka, Japan). The simulated indoor air contaminants were also prepared using a cylinder of 31 different VOCs mixed in nitrogen (Sumitomo Seika Chemicals, Osaka, Japan), as shown in Table 2.

### 2.3. Gas Sensor Analysis

**Figure 3:**
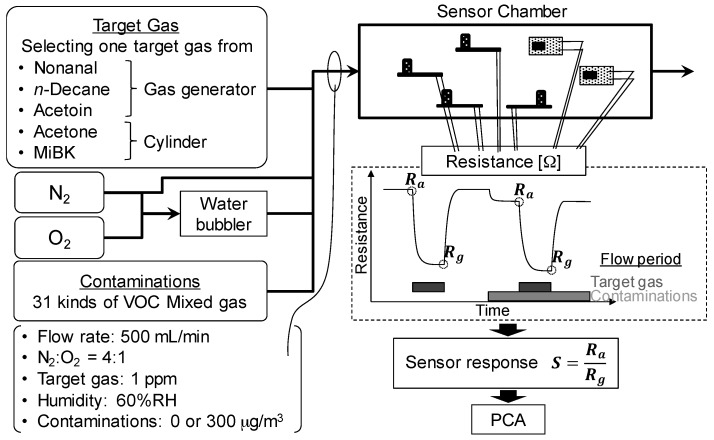
Schematic diagram of the flow apparatus for the measurement of sensor response.

The gas concentrations were measured using sensors in a flow apparatus, as shown in Figure 3. The target gas was selected from nonanal, *n*-decane, acetoin, acetone, and MiBK. Depending on the target gas, 2.5 ppm of nonanal, *n*-decane, or acetoin in nitrogen from the gas generator, or 50 ppm of acetone or MiBK in nitrogen from the cylinder, was flowed at 200 or 10 mL/min, respectively. To generate humid air, 200 mL/min of nitrogen and 100 mL/min of oxygen were introduced into a water bubbler to maintain a RH of 60%. To simulate contamination from indoor air, the mixture of 31 VOCs in nitrogen was flowed at 1.7 mL/min to maintain a total concentration of 300 μg/m^3^, which is the maximum allowed concentration of total VOCs in indoor air established by the Federal Office for Environment in Germany. Additional dry nitrogen was flowed to adjust the total flow rate to 500 mL/min. Under these conditions, the concentrations of the target gases were 1 ppm, the N_2_/O_2_ ratio was maintained at 4, and the RH was 0% or 60%.

All gas sensors were placed in a sensor chamber. The operating voltage of the heater of the four commercially available TGS sensors was 5 V, as recommended by the manufacturer, and the operating temperature of the two Pt, Pd, Au/SnO_2_ sensors (#33b and #31b) and two Zr-doped CeO_2_ sensors (No. 9 and No. 71) were maintained at 250 °C and 400 °C, respectively. The electrical resistance of the eight sensors was measured by a Graphtec Midi Logger GL200 and a Keithley 2700 digital multimeter for the TGS sensors and the Pt, Pd, Au/SnO_2_, and for the Zr-doped CeO_2_ sensors, respectively. The sensor response (*r*) is defined by Equation 1,

(1) $$r=\frac{R_{a}}{R_{g}}$$

where *R_a_* is the resistance in pure air or in simulated contaminated indoor air, and *R_g_* is the resistance in pure air or in simulated contaminated indoor air with 1 ppm of the target gases.

To select appropriate sensors from the sensor array, the difference in sensor responses (*D*) between humid air with and without contamination is also defined in Equation (2),

(2) $$D=\frac{r_{p}-r_{s}}{r_{s}}\times 100\;[\%]$$

where *r_s_* and *r_p_* are the sensor responses in humid air with and without contamination, respectively. 

### 2.4. Principal Component Analysis

The PCA was carried out using the *r* values. First, normalized scores (*x_ti_*) were calculated according to Equation (3),

(3) $$x_{ti}=\frac{\left(r_{ti}-\bar{r_{t}}\right)}{\sigma_{t}}$$

where *t* is the sensor index, *i* is the sensor response analysis index, *r_ti_* is the sensor response analysis *i* of sensor *t*, $\bar{r_{t}}$ is the average sensor response of sensor *t*, and $\sigma_{t}$ is the standard deviation of sensor *t*. Correlation coefficients (*c_ts_*) were calculated according to Equation (4),

(4) $$c_{ts}=\frac{S_{ts}}{S_{tt}S_{ss}}$$

where *s* is a sensor index and *S_ts_* is a sum of products of *s* and *t* (Equation (5)),

(5) $$S_{ts}=\sum_{i=1}^{i_{\max}}\left(r_{ti}-\bar{r_{t}}\right)\left(r_{si}-\bar{r_{s}}\right)$$

and *S_ss_* and *S_tt_* are the sum of the squares of *s* and *t* (Equation (6)).

(6) $$S_{tt}=\sum_{i=1}^{i_{\max}}(R_{si}-\bar{R_{s}}{)}^{2}$$

Eigenvalues (*λ*) were calculated according to Equation (7),

(7) |A−λE|=0

where **A** is the matrix shown in Equation (8), and **E** is the unit matrix.

(8) $$A=\left(\begin{array}{cccc} c_{11} & c_{12} & \cdots & c_{1m}\\ c_{21} & c_{22} & \cdots & c_{2m}\\ \vdots & \vdots & \ddots & \vdots \\ c_{m1} & c_{m2} & \cdots & c_{mm} \end{array}\right)$$

*c*_11_ = *c*_22_ = … = *c _mm_* = 1, *c_ts_* = *c_st_*, and *m* is the maximum number of sensors. Eigenvalues are then obtained for each sensor index (Equation (9)).

(9) $$\lambda =\lambda_{1},\lambda_{2},\cdots ,\lambda_{m}\left(\lambda_{1}>\lambda_{2}>\cdots >\lambda_{m}\right)$$

Eigenvectors of number *j* (**v_j_**) satisfy Equation (10).

(10) $$\left(A-\lambda_{j}E\right)v_{j}=0\;\left(v_{j}=(\begin{array}{c} a_{1j}\\ a_{2j}\\ \vdots \\ a_{mj} \end{array}\right))$$

Finally, PCA scores (*Z_ji_*) were evaluated according to Equation (11).

*Z_j__i_* = *a*_1*j*_*x*_1*i*_ + *a*_2*j*_*x*_2*i*_ + … + *a*_*mj*_*x_mi_* (11)

when PCA scores in principal components 1 and 2 are plotted, the coordinates of the PCA scores are (*Z*_1*i*_, *Z*_2*i*_).

In this study, we carried out the PCA at six different combinations of sensor responses, relative humidity, and indoor air contamination, as summarized in Table 3. The method for selecting gas sensors for each type of analysis is described in Section 3.1 and Section 3.2.

## 3. Results and Discussion

### 3.1. Sensor Responses and PCA in Pure Dry and Humid Air

**Figure 4:**
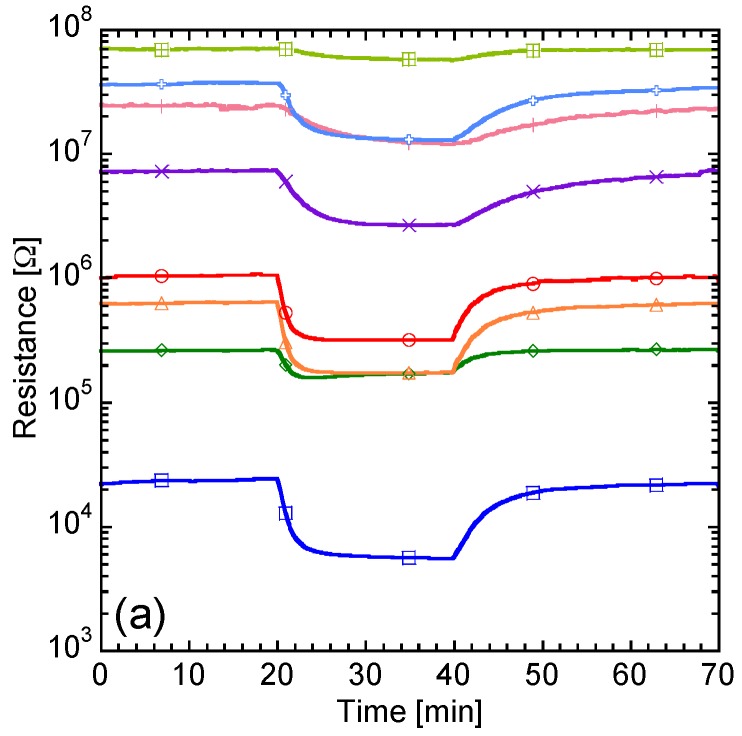

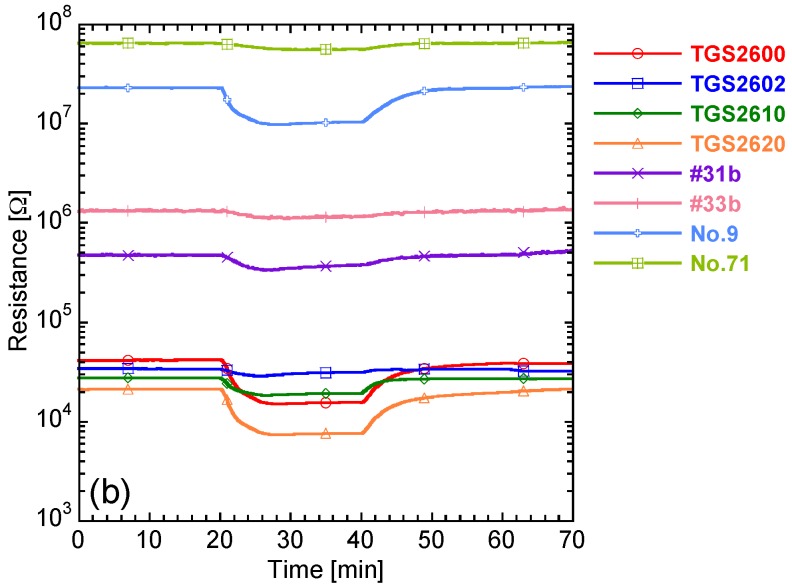
Dynamic resistance response of the six “grain boundary-response type” and two “bulk-response type” sensors to 1 ppm of acetone in (**a**) pure dry and (**b**) pure humid air.

The sensor responses (*S*) of all eight sensors at 1 ppm of the target gases were collected under pure dry and pure humid air (i.e., dry and humid air without simulated contaminated indoor air). Figure 4 shows the dynamic resistance response of the six “grain boundary-response type” (TGS2600, 2602, 2610, 2620, #31b, and #33b) and two “bulk-response type” (Nos. 9 and 71) sensors at 1 ppm of acetone in pure dry and pure humid air. Semiconductor-type gas sensors decrease in resistance in response to VOCs. In the “grain boundary-response type” sensors, oxidation of VOCs consumes oxygen adsorbed on the surface of sensor materials, decreasing the resistance of the grain boundary. In contrast, the “bulk-response type” sensors provide lattice oxygen to oxidize VOCs, and the generated surface oxygen vacancies can diffuse rapidly into the bulk because of the high diffusion coefficient for oxygen [37], decreasing the resistance of the bulk. The base resistance of the “grain boundary-response type” sensors in pure humid air was lower than that in pure dry air except for TGS2602, whereas the “bulk-response type” sensors showed almost the same base resistance in pure dry and humid air. For TGS2602, the intensity of the resistance response to acetone decreased at 60% RH because the adsorption of oxygen is prevented by the adsorption of water. Therefore, the “bulk-response type” sensors were hardly affected by the humidity, because the resistances were controlled by a different mechanism.

**Figure 5:**
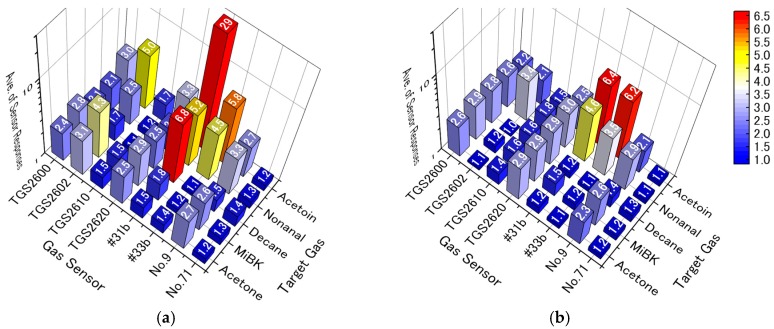
Average sensor responses of the six “grain boundary-response type” and two “bulk-response type” sensors to 1 ppm of target gases in (**a**) pure dry air and (**b**) pure humid air.

Figure 5 shows the average responses of all sensors to 1 ppm of each target gas. The average sensor responses of the six “grain boundary-response type” sensors in pure dry air have been reported previously [27], and the four commercial TGS sensors exhibit their characteristic sensor patterns in pure dry air. In the Pt, Pd, Au/SnO_2_ sensors, aldehydes were partially oxidized to acids at the working temperature (250 °C) [40]. Therefore, the two prepared Pt, Pd, Au/SnO_2_ sensors (#31b, #33b) show strong responses to easily oxidized functional groups, i.e., alcohols (acetoin) and aldehydes (nonanal), and low responses to difficult to oxidize functional groups, i.e., ketones (acetone and MiBK). Moreover, the thicker film of Pt, Pd, Au/SnO_2_ (#31b; 4.6 μm) shows stronger sensor responses to all target gases, specifically decane, as compared to a thinner film (#33b; 2.8 μm), which we have previously reported [27]. Although the two Pt, Pd, Au/SnO_2_ sensors (#31b and #33b) were both heated at 250 °C, analyzable gas flowed quickly at 500 mL/min in the sensor chamber, cooling the sensors. Therefore, the thinner film (#33b) would be cooled below the appropriate temperature. The thicker film (#31b) also shows a strong response to decane, but a weak response to acetone and MiBK, even though all of these VOCs do not have easily oxidized functional groups. Because the molecular weight of decane is larger than that of acetone and MiBK, the decomposition rate of decane would be expected to be greater than that of acetone and MiBK. 

Compared to the “grain boundary-response type” sensors, the two “bulk-response type” sensors reveal different characteristic sensor patterns. The CeZr10 single layered film (No. 9) exhibits greater sensor responses to oxygen-containing hydrocarbons (acetoin, nonanal, MiBK, and acetone) than to unoxidized hydrocarbons (decane). In the case of the CeZr10/Al_2_O_3_/Pt-CeZr10 multi-layered film (No. 71), the response to all target gases was small. However, the response to decane barely decreased with the addition of the upper layer (Pt-CeZr10), although the responses to oxygen-containing hydrocarbons decreased drastically. In the CeZr10/Al_2_O_3_/Pt-CeZr10 system, the lower CeZr10 layer works as a gas-sensing layer because of contact with the platinum comb-type electrode. The upper Pt-CeZr10 layer is then insulated from the lower CeZr10 layer by the Al_2_O_3_ layer in the middle. The upper Pt-CeZr10 layer acts as the catalytic layer. Therefore, the Pt-CeZr10 and the pure CeZr10 of the “bulk-response type” materials can easily oxidize oxygen-containing hydrocarbons, consuming them on the upper Pt-CeZr10 layer of the CeZr10/Al_2_O_3_/Pt-CeZr10 system. In contrast, decane would hardly be oxidized on the upper Pt-CeZr10 and, thus, would reach the lower CeZr10 layer. 

In pure humid air, the characteristic sensor patterns of the “grain boundary-response type” sensors changed drastically. For the four commercial TGS sensors, the response to ketones for TGS 2602 decreased drastically, and the other TGS sensors exhibited almost flat response patterns. For the two Pt, Pd, Au/SnO_2_ sensors, the characteristic strong responses to acetoin and decane from #31b decreased to almost the same response as #33b. However, the sensor response patterns of the “bulk-response type” sensors hardly changed in humid air. 

**Figure 6:**
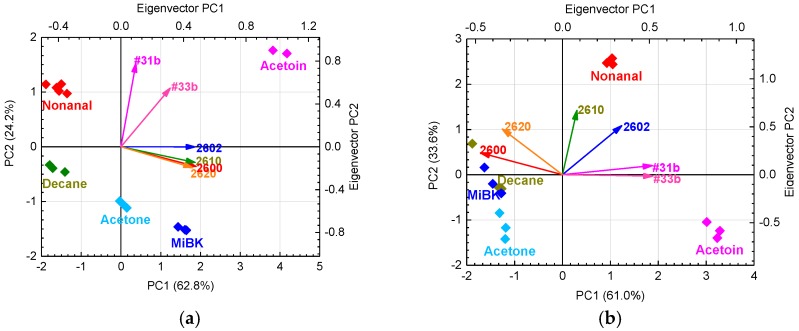

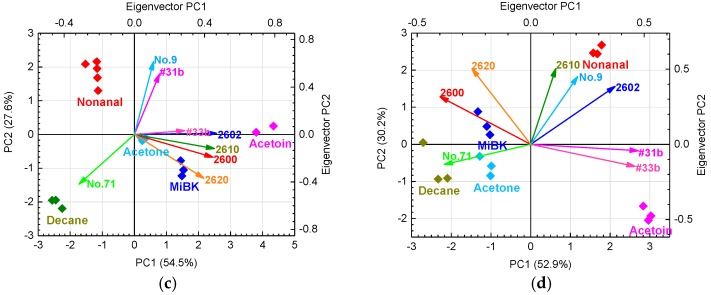
Principal component analysis (PCA) scores and eigenvectors from (**a**) six “grain boundary-response type” sensors in pure dry air; (**b**) six “grain boundary-response type” sensors in pure humid air; (**c**) six “grain boundary-response type” and two “bulk-response type” sensors in pure dry air; and (**d**) six “grain boundary-response type” and two “bulk-response type” sensors in pure humid air.

Figure 6 shows the PCA scores and eigenvectors using six (“grain boundary-response type”) or eight (“grain boundary-response type” and “bulk-response type”) sensors in pure dry and humid air. All cumulative variances of principal components (PCs) 1 and 2 in Figure 6 are over 80%. To retain the originality of the data [41], the first two PCs are normally sufficient in this study. In Figure 6a, the PCA uses sensor responses from the six “grain boundary-response type” sensors in pure dry air; the sensor response data are from the same PCA that uses the sensor responses from the six sensors in pure and contaminated dry air [27]. In dry conditions, discriminating between all target gases is possible. However, in humid conditions, the PCA scores of decane overlapped those of MiBK and were close to those of acetone. Therefore, discrimination between all gases is not possible, as shown in Figure 6b, because the response patterns from the “grain boundary-response type” sensors to decane became similar to MiBK and acetone, as shown in Figure 5b. Figure 6c and Figure 5d show the results of the PCA for the “grain boundary-response type” and “bulk-response type” sensors. The addition of sensor responses from the “bulk-response type” sensors can improve the selectivity to all target gases. In humid conditions, Figure 6d shows that the PCA scores of decane separated from those of MiBK and acetone, since the “bulk-response type” sensors keep their characteristic patterns in humid conditions, as shown in Figure 5b. Therefore, the “bulk-response type” sensors should play an important role in gas discrimination under humid conditions.

### 3.2. Sensor Responses and PCA in Humid Air with Contaminations

**Figure 7:**
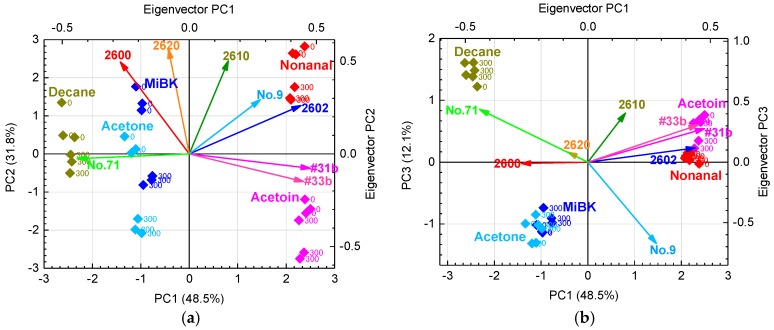
(**a**) PCA scores and PC1 and PC2 eigenvectors and (**b**) PCA scores and PC1 and PC3 eigenvectors from six “grain boundary-response type” and two “bulk-response type” sensors in humid air with 0 and 300 μg/m^3^ of indoor air VOC contaminants.

Figure 7a shows the PCA scores and eigenvectors using six “grain boundary-response type” and two “bulk-response type” sensors in humid air with 0 and 300 μg/m^3^ of VOC contamination. PC1 and PC2 showed a sufficient cumulative variance of 80.3%. The PCA scores tend to move in a negative direction parallel to the PC2 axis with contaminated indoor air, and scatter in the positive direction parallel to the PC1 axis with target gases containing easily oxidized functional groups such as alcohols (acetoin) and aldehydes (nonanal). As shown in Figure 7a, PC1 tends to be related to the functional group of the target gases. PC2 tends to be related to total organic carbon (TOC), because the PC2 score decreases with increasing concentration of indoor air contaminants. Thus, the PC2 includes information about the contaminants, but PC1 does not. Therefore, the PCA scores with PC1 and PC3 were also prepared to eliminate interference from contamination, as shown in Figure 7b. PC1 and PC3 in Figure 7b show that the PCA scores from each target gas with or without contamination are almost identical, so the PC1 and PC3 do not include the influence of contamination. As shown in Figure 7b, PC3 also tends to be related to the functional groups of the target gases. However, the PCA scores of acetone coincide with those of MiBK, and those of acetoin are also close to those of nonanal.

**Figure 8:**
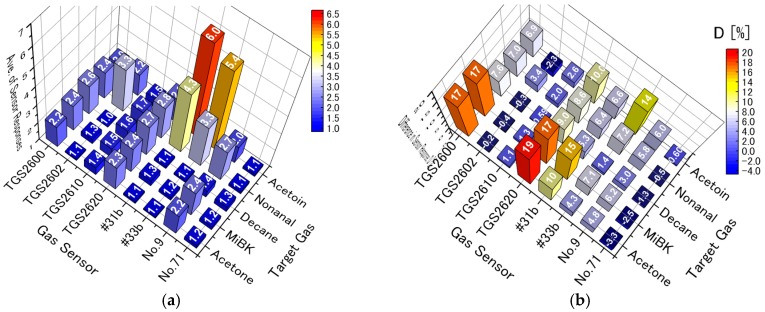
(**a**) Average sensor responses of all sensors to 1 ppm of target gases in humid air with 300 μg/m^3^ of VOC contamination; and (**b**) difference in sensor responses (*D*) between 1 ppm of target gases in pure humid air and those in humid air with 300 μg/m^3^ of VOC contamination.

As shown in Figure 7a, on the PC2 axis, the PCA scores of MiBK are larger than those of acetone, and those of nonanal are larger than those of acetoin. Therefore, the PC2 includes the molecular weight of the target gases and information about contamination. In Figure 7a, the eigenvectors of TGS 2600, 2610, and 2620 strongly contribute to PC2. As described above, PC1 and 3 in Figure 7b include barely any information about contamination. Moreover, the eigenvectors of TGS2600 and 2620 in Figure 7b are so small that they hardly contribute to the discrimination of each target gas. Figure 8 shows the average sensor responses of all sensors to 1 ppm of target gases in humid air with contamination, and the differences in sensor responses (*D*) with and without contamination. Indeed, sensors such as TGS 2600 and 2620 with large *D* are strongly affected by contamination, suggesting that their elimination from the sensor array would reduce the influence of contamination.

Moreover, the removal of one sensor from a group of sensors with high correlation coefficients has been reported to not affect the distribution of PCA scores [41]; the correlation coefficients are shown in Table 4. In addition, #31b and #33b have a strong dependence on PC1, as shown in Figure 7a. Their eigenvectors have almost the same magnitude and direction, and their high correlation coefficient of 0.99 indicates that they are highly interrelated. Consequently, we eliminated #31b because of its high correlation with #33b and TGS2600 and 2620 because of their strong responses to contamination, as discussed above. 

**Figure 9:**
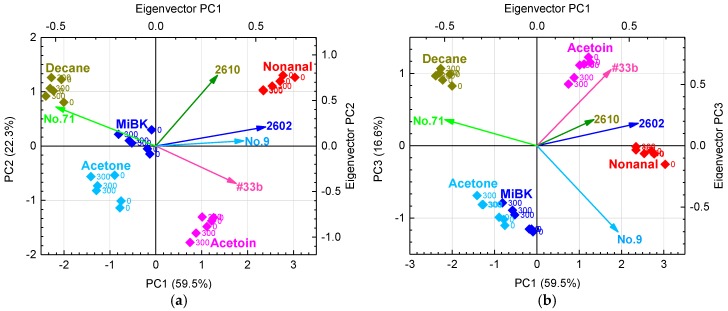
(**a**) PCA scores and PC1 and PC2 eigenvectors and (**b**) PCA scores and PC1 and PC3 eigenvectors from three “grain boundary-response type” (TGS2602, TGS2610, and #33b) and two “bulk-response type” sensors (No. 9 and No. 71) in pure humid air and humid air with 300 μg/m^3^ of VOC contamination.

Figure 9 shows the PCA scores and eigenvectors from three “grain boundary-response type” (TGS2602, TGS2610, and #33b) and two “bulk-response type” sensors (No. 9 and No. 71) in humid air with and without contamination. From PC1 and PC2 in Figure 9a, the PCA scores can discriminate all target gases and are hardly affected by the contamination. Thus, the discrimination between target gases can be improved by selecting appropriate sensors from the sensor array to eliminate unnecessary information from the total data set.

## 4. Conclusions

All eight sensors exhibit decreases in resistance in response to target VOCs. The base resistances of almost all the “grain boundary-response type” sensors in pure humid air were lower than those in pure dry air, and the intensities of their resistance responses decreased. Furthermore, the characteristic sensor patterns of all target gases decreased drastically and became nearly flat patterns. However, the “bulk-response type” sensors showed almost the same base resistance in pure dry and humid air, and the sensor patterns of the “bulk-response type” sensors hardly changed in humid air. The PCA results showed that the “bulk-response type” sensors play an important role in discriminating each target VOC in humid air. The PC2 score tends to be related to the total organic carbon (TOC) and, thus, included information related to VOC contamination, and the PC1 and PC3 scores tend to be related to the functional groups of the target gases. From PC1 and PC2, we removed sensors with large contributions to VOC contaminations, i.e., large differences in sensor responses (*D*) between pure humid air and pure humid air with 300 μg/m^3^ of VOC contamination, to reduce the effects of contamination on the sensor array. Consequently, selectivity for the target gases was maintained, and the VOC contamination did not greatly affect the measurements.

## Figures and Tables

**Table 1:** Details of the four TGS, two Pt, Pd, Au/SnO_2_, and two Zr-doped CeO_2_ sensors used in this study.

Name of Sensor	Detected Compounds and Sensor Details
TGS 2600	H_2_ and alcohol (manufactured by Figaro)
TGS 2602	Alcohol, ammonia, VOC, and H_2_S (manufactured by Figaro)
TGS 2610	Liquefied petroleum gas (manufactured by Figaro)
TGS 2620	Alcohol and solvent vapors (manufactured by Figaro)
#31b	VOCs (Pt, Pd, Au/SnO_2_; film thickness: 4.6 μm)
#33b	VOCs (Pt, Pd, Au/SnO_2_; film thickness: 2.8 μm)
No. 9	Oxygen and VOCs (CeZr10; film thickness: 9 μm)
No. 71	Oxygen and VOCs (CeZr10/Al_2_O_3_/Pt-CeZr10; film thickness: 9 μm/4 μm/9 μm)

**Table 2:** Concentrations and functional group classifications of each component in the mixture of 31 kinds of volatile organic compounds (VOCs) in nitrogen.

Group	Component	Concentration in Cylinder [ppm]
Aromatic Hydrocarbon	Benzene	0.52
	Toluene	0.50
	*o*-Xylene	0.50
	*m*-Xylene	0.50
	Styrene	0.50
	Ethylbenzene	0.50
	*n*-Propylbenzene	0.49
	1,2,3-Trimethylbenzene	0.50
	1,2,4-Trimethylbenzene	0.50
	1,3,5-Trimethylbenzene	0.50
	*o*-Ethyltoluene	0.50
Aliphatic Hydrocarbon	*n*-Hexane	0.50
	2-Methylpentane	0.50
	3-Methylpentane	0.50
	*n*-Heptane	0.50
	2,4-Dimethylpentane	0.51
	*n*-Octane	0.50
	2,2,4-Trimethylpentane	0.50
	*n*-Nonane	0.50
	*n*-Decane	0.50
	*n*-Undecane	0.50
	*n*-Dodecane	0.100
	Methylcyclopentane	0.50
	Cyclohexane	0.50
	Methylcyclohexane	0.50
Terpene	*α*-Pinene	0.50
	*β*-Pinene	0.51
	(+)-Limonene	0.50
Halide	*p*-Dichlorobenzene	0.50
Ester	Butyl acetate	0.99
Ketone	Methyl *i*-butyl ketone	2.99
**Total**		18.11 *

* 18.11 [ppm] = 8.92 × 10^4^ [μg/m^3^].

**Table 3:** Selection of sensor responses in the six types of analysis.

	Sensors								Humidity [%RH]	Contamination [μg/m^3^]	Results
	TGS 2600	TGS 2602	TGS 2610	TGS 2620	#31b	#33b	No. 9	No. 71			
Type I	v	v	v	v	v	v	-	-	0	0	Figure 6a
Type II	v	v	v	v	v	v	-	-	60	0	Figure 6b
Type III	v	v	v	v	v	v	v	v	0	0	Figure 6c
Type IV	v	v	v	v	v	v	v	v	60	0	Figure 6d
Type V	v	v	v	v	v	v	v	v	60	0, 300	Figure 7
Type VI	v	-	-	v	-	v	v	v	60	0, 300	Figure 9

* “v” means that the sensor responses (*r*) from the sensor were used.

**Table 4:** Correlation coefficients of the eight sensors.

Sensor	TGS 2600	TGS 2602	TGS 2610	TGS 2620	#31b	#33b	No. 9	No. 71
TGS2600	1							
TGS2602	−0.19	1						
TGS2610	0.41	0.70	1					
TGS2620	0.84	0.18	0.58	1				
#31b	−0.56	0.75	0.24	−0.19	1			
#33b	−0.60	0.64	0.13	−0.26	0.99	1		
No. 9	0.053	0.62	0.33	0.22	0.26	0.15	1	
No. 71	0.38	−0.68	−0.056	0.065	−0.72	−0.68	−0.74	1

## References

[1] Wang C., Mbi A., Shepherd M. (2010). A Study on Breath Acetone in Diabetic Patients Using a Cavity Ringdown Breath Analyzer: Exploring Correlations of Breath Acetone with Blood Glucose and Glycohemoglobin A1C. IEEE Sens. J..

[2] Poli D., Carbognani P., Corradi M., Goldoni M., Acampa O., Balbi B., Bianchi L., Rusca M., Mutti A. (2005). Exhaled volatile organic compounds in patients with non-small cell lung cancer: Cross sectional and nested short-term follow-up study. Respir. Res..

[3] Fuchs P., Loeseken C., Schubert J.K., Miekisch W. (2010). Breath gas aldehydes as biomarkers of lung cancer. Int. J. Cancer.

[4] Song G., Qin T., Liu H., Xu G.B., Pan Y.Y., Xiong F.X., Gu K.S., Sun G.P., Chen Z.D. (2010). Quantitative breath analysis of volatile organic compounds of lung cancer patients. Lung Cancer.

[5] Amann A., Spanel P., Smith D. (2007). Breath analysis: The approach towards clinical applications. Mini Rev. Med. Chem..

[6] Peng G., Tisch U., Adams O., Hakim M., Shehada N., Broza Y.Y., Billan S., Bortnyak R.A., Kuten A., Haick H. (2009). Diagnosing lung cancer in exhaled breath using gold nanoparticles. Nat. Nanotech..

[7] Westhoff M., Litterst P., Freitag L., Urfer W., Bader S., Baumbach J.-I. (2009). Ion mobility spectrometry for the detection of volatile organic compounds in exhaled breath of patients with lung cancer: Results of a pilot study. Thorax.

[8] Mazzone P.J., Hammel J., Dweik R., Na J., Czich C., Laskowski D., Mekhail T. (2007). Diagnosis of lung cancer by the analysis of exhaled breath with a colorimetric sensor array. Thorax.

[9] Herberger S., Herold M., Ulmer H., Burdack-Freitag A., Mayer F. (2010). Detection of human effluents by a MOS gas sensor in correlation to VOC quantification by GC/MS. Build. Environ..

[10] Yang J.-W., Cho H.-J., Lee S.-H., Lee J.-Y. (2004). Characterization of SnO_2_ ceramic gas sensor for exhaust gas monitoring of SVE Process. Environ. Monit. Assess..

[11] Wolfrum E.J., Peterson R.M.M.D., Sluiter J. (2006). Metal oxide sensor arrays for the detection, differentiation, and quantification of volatile organic compounds at sub-parts-per-million concentration levels. Sens. Actuators B Chem..

[12] Szczurek A., Maciejewska M., Flisowska-Wiercik B., Bodzoj L. (2009). Application of a sensor system for determining the kind and quantity of two component VOC mixtures in air after the use of solvents. J. Environ. Monit..

[13] Wolfrum E.J., Meglen R.M., Peterson D., Sluiter J. (2006). Calibration transfer among sensor arrays designed for monitoring volatile organic compounds in indoor air quality. IEEE Sens. J..

[14] Itoh T., Matsubara I., Kadosaki M., Sakai Y., Shin W., Izu N., Nishibori M. (2010). Effects of High-Humidity Aging on Platinum, Palladium, and Gold Loaded Tin Oxide—Volatile Organic Compound Sensors. Sensors.

[15] Itoh T., Matsubara I., Nishibori M., Izu N., Shin W. (2012). Calibration Gas Preparation for Non-Disposable Portable MOx, PID, and IER VOC Detectors. Sens. Lett..

[16] Manorama S., Devi G.S., Rao V.J. (1994). Hydrogen sulfide sensor based on tin oxide deposited by spray pyrolysis and microwave plasma chemical vapor deposition. Appl. Phys. Lett..

[17] Shimura M., Yasuno Y., Iwakura M., Shimada Y., Sakai S., Suzuki K., Sakamoto S. (1996). A new monitor with a zinc-oxide thin film semiconductor sensor for the measurement of volatile sulfur compounds in mouth air. J. Periodontol..

[18] Tanda N., Washio J., Ikawa K., Suzuki K., Koseki T., Iwakura M. (2007). A new portable sulfide monitor with a zinc-oxide semiconductor sensor for daily use and field study. J. Dent..

[19] Itoh T., Taguchi Y., Izu N., Matsubara I., Nakamura S., Suzuki K., Kanda K., Shin W., Nishibori M. (2011). Alternating Current Impedance Analysis of CeO_2_ Thick films as Odor Sensors. Sensors.

[20] Byun H.-G., Persaud K.C., Pisanelli A.M. (2010). Wound-State Monitoring for Burn Patients Using E-Nose/SPME System. ETRI J..

[21] Yu J.-B., Byun H.-G., Zhang S., Do S.-H., Lim J.-O., Huh J.-S. (2011). Exhaled Breath Analysis of Lung Cancer Patients Using a Metal Oxide Sensor. J. Sens. Sci. Technol..

[22] Hossein-Babaei F., Amini A. (2012). A breakthrough in gas diagnosis with a temperature-modulated generic metal oxide gas sensor. Sens. Actuators B Chem..

[23] Dymerski T., Gębicki J., Wiśniewska P., Śliwińska M., Wardencki W., Namieśnik J. (2013). Application of the Electronic Nose Technique to Differentiation between Model Mixtures with COPD Markers. Sensors.

[24] Itoh T., Nakashima T., Akamatsu T., Izu N., Shin W. (2013). Nonanal gas sensing properties of platinum, palladium, and gold-loaded tin oxide VOCs sensors. Sens. Actuators B Chem..

[25] Sakai Y., Kadosaki M., Matsubara I., Itoh T. (2009). Preparation of total VOC sensor with sensor-response stability for humidity by noble metal addition to SnO_2_. J. Ceram. Soc. Jpn..

[26] Zaromb S., Stellar J.R. (1984). Theoretical basis for identification and measurement of air contaminants using an array of sensors having partially overlapping sensitivities. Sens. Actuators.

[27] Itoh T., Akamatsu T., Izu N., Shin W., Byun H.-G. Monitoring of disease-related volatile organic compounds in simulated room air. Proceedings of the 2014 IEEE Sensors.

[28] Cesare F.D., Pantalei S., Zampetti E., Macagnano A. (2008). Electronic nose and SPME techniques to monitor phenanthrene biodegradation in soil. Sens. Actuators B Chem..

[29] Natale C.D., Macagnano A., Martinelli E., Paolesse R., D’Arcangelo G., Roscioni C., Finazzi A., D’Amico A. (2003). Lung cancer identification by the analysis of breath by means of an array of non-selective gas sensors. Biosens. Bioelectron..

[30] European Commission Joint Research Centre Environment Institute (1997). Indoor Air Quality and Its Impact on Man—Report No. 19: Total Organic Compounds (TVOC) in Indoor Air Quality Investigations.

[31] Ministry of Health, Labour and Welfare Japan (1999). Midterm Report of Sick Building Syndrome Committee—Parts 6 and 7.

[32] ISO 16000-6 (2004). Indoor Air—Part 6: Determination of Volatile Organic Compounds in Indoor and Test Chamber Air by Active Sampling on Tenax TA Sorbent, Thermal Desorption and Gas Chromatography Using MS/FID.

[33] Kadosaki M., Sakai Y., Tamura I., Matsubara I., Itoh T. (2008). Development of oxide semiconductor thick film gas sensor for the detection of total volatile organic compounds. Electron. Commun. Jpn..

[34] Yamazoe N., Shimanoe K. (2008). Theory of power laws for semiconductor gas sensors. Sens. Actuators B Chem..

[35] Yamazoe N., Shimanoe K. (2009). New perspectives of gas sensor technology. Sens. Actuators B Chem..

[36] Izu N., Shin W., Matsubara I., Murayama N. (2004). Development of Resistive Oxygen Sensors Based on Cerium Oxide Thick Film. J. Electroceram..

[37] Izu N., Shin W., Matsubara I., Murayama N. (2006). Evaluation of response characteristics of resistive oxygen sensors based on porous cerium oxide thick film using pressure modulation method. Sens. Actuators B Chem..

[38] Itoh T., Izu N., Akamatsu T., Shin W., Miki Y., Hirose Y. (2015). Elimination of Flammable Gas Effects in Cerium Oxide Semiconductor-Type Resistive Oxygen Sensors for Monitoring Low Oxygen Concentrations. Sensors.

[39] Itoh T., Uchida T., Matsubara I., Izu N., Shin W., Miyazaki H., Tanjo H., Kanda K. (2007). Preparation of γ-alumina large grain particles with large specific surface area via polyol synthesis. Ceram. Int..

[40] Itoh T., Lee D., Goto T., Akamatsu T., Izu N., Shin W., Kasuga T. (2016). Analysis of Recovery Time of Pt- Pd- and Au-Loaded SnO_2_ Sensor Material with Nonanal as Large-Molecular-Weight Volatile Organic Compounds. Sens. Mater..

[41] ASrivastava K., Dravid V.P. (2006). On the performance evaluation of hybrid and mono-class sensor arrays in selective detection of VOCs: A comparative study. Sens. Actuators B Chem..

